# Impact of smartphone addiction on depression and self‐esteem among nursing students

**DOI:** 10.1002/nop2.506

**Published:** 2020-05-19

**Authors:** Sayeda Mohamed Mohamed, Mona Hamdy Mostafa

**Affiliations:** ^1^ Psychiatric Mental Health Nursing Department Faculty of Nursing Cairo University Cairo Egypt

**Keywords:** depression, nursing students, self‐esteem, smartphone addiction

## Abstract

**Aim:**

To assess the impact of smartphone addiction on depression and self‐esteem among nursing students.

**Setting:**

Faculty of Nursing.

**Design:**

Cross‐sectional, survey‐based research design used in this study. Sample: Stratified random sample consists of 320 nursing students. Tools: Four tools used for data collection: personal data sheet, Hamilton rating scale of depression, smartphone addiction scale and Self‐esteem Inventory.

**Results:**

About 95.8% of nursing students reported smartphone addiction, while 32.5% were pseudonormal of depression. Twenty‐eight percent of them had low self‐esteem. The study also revealed a statistically significant positive correlation between smartphone addiction and levels of depression. There was a statistically significant negative correlation between levels of depression and self‐esteem.

## 1 INTRODUCTION

The introduction of smartphones has revolutionized many aspects of daily living practice. They are multifunctioning, hand‐held devices that can combine the functions of both computers and telephones. Since the first smartphone was launched in 2007, these devices become more capable of performing different tasks, including, but not limited to, document editing, entertaining, chatting and online shopping. Additionally, the smartphone is a crucial tool for rapid and easy access to a wide range of information via the Internet (Dikeç & Kebapçı, 2018).

Previous epidemiological figures highlighted that up to 80% of students use smartphones daily, with median daily hours of 5–7 hr (Dikeç & Kebapçı, 2018 and Kibona & Mgaya, 2015). The main uses of smartphones among students were messaging (98.1%), social networking (91.6%), visiting websites (89.7%) and playing games (84.1%) (Qader & Omar, 2015). The use of smartphones for social activities, the primary access to a social network, was reported to be notably higher than its use for education activities (Kibona & Mgaya, 2015).

Recently, a growing body of published literature raises concerns about the overuse of smartphones by students. Smartphone addiction is defined as a statistically significant distribution in routine daily‐life practices due to the overuse of smartphones (Soni, Upadhya, & Jain, 2017).

Broadly, smartphone addiction is one form of behavioural addiction. Many central components of addiction have been established by behavioural addictions, including salience, mood change, preoccupation, tolerance, lack of control, withdrawal symptoms, lies, excessive use and loss of interest, interpersonal and intrapersonal conflict and relapse. Behavioural addiction can be considered similar to substance addiction and many aspects such as functional impairment and tremendous difficulties in withdrawal (Mitchell & Hussain, 2018).

Smartphone addiction is a significant public health concern with a significant impact on the mental and behavioural status of its users. Previous evidence has linked smartphone addiction to impaired behavioural attitude, low school/work performance, impaired social interaction and relationship difficulties. Physically, the overuse of smartphones was linked to higher risks of musculoskeletal pain, headache, blurred vision and hearing impairment (Soni et al., 2017).

Smartphone addictive people tend to feel depressed and isolated without their smartphones; besides, they can experience other symptoms of addiction such as preoccupation, tolerance, lack of control, withdrawal, mood modification, conflict, lies, excessive use and loss of interest. Depression and low self‐esteem are general reflections of psychological well‐being, which are believed to be highly correlated with smartphone addiction (Alhassan et al., 2018).

Cognitive behavioural therapy, motivational interviewing, music therapy, art therapy and exercises may be used to treat smartphone dependency. Exercise is already used to combat anxiety and depression; its use against smartphone addiction not only increases people's self‐confidence and happiness but can also treat patients with poor posture or carpal tunnel syndrome due to addiction **(**Dikeç & Kebapçı, 2018).

A multidisciplinary approach is crucial in the prevention and management of addiction, and nurses play a decisive part in this approach. Thus, nurses should be aware of all aspects related to the physical and mental consequences of behavioural addictions and basic principles for their treatment **(**Dikeç & Kebapçı, 2018).

Addictions nurses should benefit from protective measures for the prevention and effects of addiction not only in the clinic but also in adolescent schools and facilities wherever they can access them. They can build preventive strategies, taking into account the evidence from future studies concerning university students, and help to raise awareness concerning the behavioural addictions and their adverse effects (Kim, 2013).

### 1.1 Significance of the study

Smartphones have become highly accessible devices. Adolescents are one of the most vulnerable groups for smartphone addiction owing to their better dealing with technological advances and more inadequate impulse control compared with adults. While the physical consequences of the overuse of smartphones can be easily diagnosed and managed, mental health issues are statistically significant concerns with smartphones overuse. Withdrawal, preoccupation, tolerance, lack of control, mood modification, conflict, lies, excessive use and loss of interest are reported among students with smartphone addiction. Depression and low self‐esteem can be devastating complications of smartphone addiction, especially among vulnerable groups.

Previous reports demonstrated a higher degree of depressive symptoms among students with smartphone addiction. Also, smartphone users noted to suffer from low self‐esteem. Mental health nurses must recognize those students who suffer from severe psychological problems resulting from smartphone addiction. Because of the increasing prevalence of using modern technology, there is a need to focus on depression and self‐esteem among nursing students, quantify severity and develop proper coping methods to deal with depressive and negative emotions. There are scanty of Egyptian studies that shed light on screening the impact of smartphone addiction among nursing students.

Therefore, the current study aimed to fill the gap with understanding the extent of smartphone addiction among adolescents about the presence of depression and low self‐esteem among nursing students with smartphone addiction. Moreover, the results of this study will assist the mental health nurses in picking out the appropriate methods to intervene effectively with students suffering from smartphone addiction. Therefore, it is necessary to equip academic staff working with nursing students and family counselling become crucial for providing care for nursing students using smartphone. Thus, the current study aimed to assess the impact of smartphone addiction on depression and self‐esteem among this group.

### 1.2 Aim of the study

The current study aimed to assess the impact of smartphone addiction on depression and self‐esteem among nursing students and Faculty of Nursing.

#### 1.2.1 Research questions

1. What is the impact of smartphone addiction on depression and self‐esteem among nursing students?
2. Is there a relationship between smartphone addiction, depression and self‐esteem among nursing students?

## 2 METHODS

### 2.1 Research design

The cross‐sectional survey‐based research design was used in the current study.

### 2.2 Setting

The study was carried out at the Faculty of Nursing.

### 2.3 Sample

A stratified random sample of 320 nursing students participated in this study. The sample size was calculated using G‐power version 3.3.1 with a power of β = 1‐0.95, with a significance level of 0.05 (two tails) and a medium effect size of 0.3.

#### 2.3.1 Sampling technique

After obtaining a list of all the students enrolled in the Faculty of Nursing, Cairo University, a total of 1600 students underwent stratified randomization according to the level of education. The statistical analyser determined the number of students at each level: first level (96), the second level (105), the third level (69) and the fourth level (50). Random samples were then selected from each stratum (level of education) using a random table technique.

### 2.4 Tools

Four scales were used in the current study:

1. Personal data sheet was constructed by the researchers. It includes information about the participants such as gender, academic level and economic status, type of residence, smoking and the number of hours using smartphones.
2. Hamilton Rating Scale of Depression was developed by Hamilton (1960–1967). It was used to identify the result of an interview to facilitate the measurement of factors such as depth of depression; it was translated into Arabic by Fatten (1997). It is composed of 24 items. Each item is scored from 0 to 2) or from 0 to 4. The severity of depression was classified according to the resulting score into normal (0–9), pseudonormal (10–17), mild depression (18–25), moderate depression (26–28) and severe depression (29–50). The rating scale was completed in 15 to 20 min. The reliability of the scale was measured by inter‐rater reliability of 0.82 to 0.98, and test–retest reliability of 0.81 to 0.98 of this scale in this study was *r* = .81.
3. Smartphone addiction scale (SAS) was developed by Kwon, Kim, Cho, & Yang (2013). The scale was considered an appropriate tool for assessing smartphone addiction. The SAS consists of 33 items. All items were answered using a 5‐point Likert scale format ranging from strongly disagree (a), disagree (b), somewhat (c), agree (d), to strongly agree (e). The scale was divided into six subscales: daily‐life disturbance (5 items), positive anticipation (7 items), withdrawal (4 items), cyberspace‐oriented relationship (4 items), overuse (8 items) and tolerance (5 items). Kwon et al. (2013) SAS subscales have been described as follows: "Daily‐life disturbance" explains the challenge of focusing class, skipping scheduled assignments and suffering from symptoms such as neck pain, lightheadedness or sleep. "Positive anticipation" describes stress reduction by using the smartphone and feeling empty when no smartphone exists. "Withdrawal" describes the feelings of impatience, anxiety and intolerability when there is no smartphone. "Cyberspace‐oriented relationship" defines more close relationships on social networking with friends than in real life. "Overuse" means uncontrollable use of the smartphone. "Tolerance" is defined as always attempting to control smartphone use but not being effective. The total SAS score can be calculated, which varies from 33–165. Score 156 or more indicates a higher smartphone addiction level.
4. Self‐esteem Inventory is a self‐report questionnaire that was developed by Cooper Smith (1981). It measures attitudes towards the self in a variety of areas (family, peers, school and general social activities) for adolescents and adults. It consisted of 39 items. The Arabic version of this tool was translated by El Didan (2003). It was categorized under four subscales: self‐esteem by family (9 items); self‐esteem by the school (10 items); self‐esteem by peer (10 items); and self‐esteem by general (10 items). The tool is on a 5‐point Likert scale, ranging from 5 (strongly agree) to 1 (strongly disagree). The total score is ranging from 39–195. High scores indicate high self‐esteem among students. Three experts checked the content validity of these questionnaires in the field of mental health nursing and statistics.

### 2.5 Ethical considerations

Official approval was obtained from the Vice‐Dean for undergraduate students and education. After the eligible participants were identified, they informed that they have the right to withdraw from the research at any time without giving any reason. Informed consent was obtained from all eligible participants who agreed to participate in the study. Data confidentiality and students' privacy were secured. The researchers created code numbers and kept them to keep the students anonymous.

### 2.6 Procedure

Official approval was obtained from the Vice‐Dean of Education and Student Affairs at the Faculty of Nursing and also from head of different departments at the Faculty of Nursing to access the potential participants, and the researchers approached the available sample to identify the eligible participants for the current study. The researchers started to contact students who met the inclusion criteria of the study. Then, the researchers interviewed all students who met inclusion criteria and agreed to participate in the study. The aim of the study was explained to the selected students. Written consent was obtained from the study participants. The researchers began data collection by introducing themselves to the participants and explained the content of the study tools to establish initial rapport and gain cooperation between students and researchers. All questions related to the study tools were answered, and a detail explanation was given to participants.

### 2.7 Pilot study

A pilot study was carried out on 30 students, who were excluded from the actual sample, to ensure the clarity and applicability of the study measures. No modifications were needed to test the feasibility and applicability of the study tools. Participants who participated in the pilot study were excluded later from the actual study.

### 2.8 Statistical design

Statistics was analysed using the Statistical Package for the Social Sciences (SPSS), version 21. Frequency and percentage were used for numerical data, and mean and standard deviation. For parametric analysis, *t* test and ANOVA (analysis of variance) were used. The Pearson correlation test was used to determine the correlation between smartphone addiction and both self‐esteem and depression.

## 3 RESULTS

Table 1 presents the personal data of 320 nursing students. The nursing student sample consisted of 54.7% female students. Also, about one‐third of the sample (32.8%) was in the second level of education, while the minority (15.6%) of nursing students were in the fourth level of education. As regards the number of hours using a smartphone, the highest frequency of the number of hours using smartphone daily was 6–10 hr (40.9%), while the primary purpose of its use was playing games, watching films, and others (45.9%).

**Table 1:** Frequency distribution of personal data of nursing students (*n* = 320 students)

Variables	Nursing students
	No. %
Gender	
Male	145 (45.3%)
Female	175 (54.7%)
Level of education	
First level	96 (30%)
Second level	105 (32.8%)
Third level	69 (21.6%)
Fourth level	50 (15.6%)
Number of hours using smartphone	
1–5 hr	102 (31.9%)
6–10 hr	131(40.9%)
11–15	55 (17.1%)
16–20	22 (7.1%)
21–24	9 (2.8%)
Purpose of using smartphone	
Calling + Study	29 (9.1%)
Social media	143 (44.7%)
Playing games, watching films and others	147 (45.9%)

### 3.1 Research question 1

Table 2 reveals that most nursing students either agrees or strongly agrees that they were addicted to smartphone (95.8%). There is a high percentage of nursing students who strongly agree that they were addicted to smartphone in the daily‐life disturbance domain (73.8%), withdrawal domain (74%), overuse domain (69.7%), tolerance domain (67.8%), cyberspace‐oriented relation domain (62.5%) and positive anticipation domain (59.7%).

**Table 2:** Smartphone addiction levels among nursing students (*n* = 320 students)

Smartphone addiction scale	Strongly disagree		disagree		Neither agree nor disagree		Agree		Strongly agree		% of respondents’ agreement
	No.	%	No.	%	No.	%	No.	%	No.	%	%
1. Daily‐life disturbance	0	0	0	0	11	3.4	73	22.8	236	73.8	96.6%
2. Positive anticipation	0	0	0	0	8	2.5	121	37.8	191	59.7	97.5%
3. Withdrawal	0	0	0	0	12	3.8	71	22.2	237	74	96.2%
4. Cyberspace‐oriented relation	0	0	0	0	22	6.9	98	30.6	200	62.5	93.1%
5. Overuse	0	0	0	0	6	1.9	91	28.4	223	69.7	98.1%
6. Tolerance	0	0	1	0.3	16	5	86	26.9	217	67.8	94.7%
Total	0	0	1	0.3	13	4.6	90	28.	217	67.8	95.8%

Table 3 describes the levels of depression among nursing students; most participants were normal (53.8%). One‐third of the studied sample (32.5) was in the pseudonormal level of depression, while other levels of depression were reported in the remaining students (13.7%).

**Table 3:** Distribution of depression levels among nursing students (*n* = 320 students)

Depression levels	No.(%)
Normal	172 (53.8%)
Pseudonormal	104 (32.5%)
Mild	17 (5.3%)
Moderate	22 (6.8%)
Severe	5 (1. 6%)

Table 4 illustrates that about one‐third of the nursing students had disagreed level of total self‐esteem (28%). The highest reported responses were agreed (29%) in the family domain, somewhat (30%) in the school domain, disagreed (39) in the peer domain and disagreed (33%) in the general domain.

**Table 4:** Self‐esteem domains among nursing students (*n* = 320 students)

Self‐esteem domains	Strongly disagree		disagree		Somewhat		agree		Strongly agree	
	No	%	No	%	No	%	No	%	No	%
Self‐esteem by family	49	15	57	18	70	22	92	29	51	16
Self‐esteem by school	48	15	69	21	96	30	71	22	37	11
Self‐esteem by peer	30	9	124	39	77	24	64	20	26	8
Self‐esteem in general	27	9	107	33	79	25	78	24	29	9
Total	38	12	90	28	81	25	76	24	35	11

### 3.2 Research question 2

Table 5 illustrates that there was a statistically significant positive correlation between smartphone addiction and levels of depression (*r* = .996, *p* = .000) and statistically significant negative correlation between levels of self‐esteem and smartphone addiction (*r* = −.329, *p* = .000). There was a negative correlation between levels of depression and levels of self‐esteem (*r* = −.921, *p* = .006).

**Table 5:** Correlation matrix of nursing students in relation to the studied variables (*n* = 320 students)

Variables	Smartphone addiction	Depression	Self‐esteem
Smartphone addiction	1		
Depression	*R* = .996	*p* = .000	1
Self‐esteem	*R* = −.329	*R* = −.921	1
	*p* = .000	*p* = .006	

r = pearson correlation; P = level of significance.

## 4 DISCUSSION

The results of this study showed that most nursing students were female students and they were at the second level of education. This result could be interpreted by the increase in the number of female students in four levels of education at the Faculty of Nursing. This finding was congruent with that of the recent studies carried out by Dikeç and Kebapçı (2018) and Lee, Jin‐Kim, Choi, and Yoo (2018), which showed that most participants were female (91.4%) and younger than 25 years old (98.1%). There were 83 freshmen (25.6%), 73 sophomores (22.5%), 87 juniors (26.9%) and 81 seniors (25.0%).

As regards the number of hours using smartphones, the current study showed that nearly half of the participants used a smartphone daily for between 6–10 hr and nursing students used a smartphone for social media. This result may be due to various forms of social media, which as used as tools enabling nursing students to engage in communication, obtaining information, sharing their status, uploading videos or pictures and chatting in groups. Besides, quick texting with applications can be downloaded on phones. This finding was consistent with that of the Turkish Statistical Institute (TUIK) (2015), which reported that social media constituted 78.8% of smartphone usage in 2014.

As regards the levels of smartphone addiction, smartphone addiction subscales in this study were high among nursing students. This finding may be due to individuals who are stuck in a vicious cycle. This finding was in agreement with that of the previous study carried out by Kwon and Paek (2016), which showed that students tend to use the smartphone more and are inclined to develop a smartphone addiction. Another study showed that up to 85.4% of students had a high index of smartphone use (Sethuraman, Rao, Charlette, Thatkar, & Vincent, 2018).

Boumosleh and Jaalouk (2017) found that a considerable proportion of students reported indications of withdrawal indications of tolerance. Also, Ganganahalli, Tondare, and Durgawale (2014) reported during examination days, nearly 90% of students felt uncomfortable and disconnected after the lack of smartphone use for hours.

As regards the prevalence of depression among the studied sample, we found that about one‐third of students with smartphone addiction had pseudonormal levels of depression. This result could be interpreted as smartphone addiction can be a predisposing factor to depression, either indirectly or through a mediating effect. The nursing students face many stressors during their educational experiences such as clinical setting environment, death and dying patients, examinations, curriculum and academic workload and lack of free recreational time; all of these factors can precipitate depression. This result is in agreement with the study conducted by Kim et al. (2019), who reported that the prevalence of depression increased significantly the odds meeting criteria for smartphone addiction.

Concerning self‐esteem, the current research revealed that about one‐third of nursing students disagreed about their self‐esteem. This finding could be interpreted as nursing students require self‐esteem as a future health professional who has to look after individuals' health. This finding was congruent with that of the study carried out by Oh (2017), which showed that self‐esteem significantly contributed to smartphone addiction.

The current study revealed a statistically significant difference between smartphone addiction and gender. This finding may be interpreted as male students were more likely to have a habit of playing games more often than female students and watching mobile phone videos, phone call or texting was also observed in higher proportion in male students, whereas female students were more inclined to use the mobile phone communication functions and social networking services.

This finding was congruent with that of the study carried out by Kalyani, Reddi, Ampalam, Kishore, and Elluru (2019), which showed that smartphone addiction was present in 37.3% in male students and 24.5% in female students. This finding was inconsistent with that of the study carried out by Demirci, Akgonul, and Akpinar (2015), which showed that smartphone addiction was higher in female students; the study also showed that there is not much of gender variation in the prevalence of smartphone addiction in male students (30.3%) and female students (29.3%)

The current study revealed no statistically significant difference between smartphone addiction and the number of hours and the purpose of using a smartphone. This finding may be due to increased time spent using a smartphone that may lead to problematic use as difficulties in the expression of emotion, higher interpersonal anxiety, depression and low self‐esteem. The result was in agreement with Alhassan et al., (2018), who found that the time spent using a smartphone was significantly related to problematic smartphone use, which causes anxiety, irritability, mood swings and sadness.

Also, the current finding was congruent with that of the study carried out by Boumosleh and Jaalouk,(2017), which showed that texting (83%), entertainment/calling family members (67%) and calling friends (62%) were the top three reasons for smartphone use. Similarly, the study carried out by Süt, Kurt, Uzal, and Özdilek (2016) showed that students attending the health sciences faculty have the highest rate of reasons for smartphone use, that is 56.8% for "connecting to social networks." Almost nearly two‐thirds of current study participants decided to some extent that they would constantly check their phones so as not to miss other people's conversations. Overusage of smartphones can impair the hand function by causing pain in the thumb and lowering the pinch strength (İNal, Çetİntürk, Akgönül, & Savaş, 2015).

In this present study, there was a positive correlation between smartphone addiction and depression. Smartphone addiction levels are increasing with depression levels. This result could be interpreted as smartphone use is now the pressure of daily obligations from work, school and personal life. Smartphone use and demands for achievement were also identified as direct sources of stress depression. This finding was supported by Oh (2017) revealed that, the relationships between nursing student's Smartphone addiction are higher than, as well as self‐esteem was lower.

This result is consistent with that of the previous study carried out by Kim et al. (2015), which showed that depressive people tend to use the smartphone more and are inclined to develop a smartphone addiction as a comorbidity of depression in smartphone addiction. Thus, identifying students with depression and providing them with appropriate supportive services may help them to prevent addiction to a smartphone (Kwon & Paek, 2016).

Concerning self‐esteem, this current study illustrates the nursing student's low percentage of self‐esteem relationship between depression and self‐esteem. This finding could be interpreted as smartphone addiction affects nursing students, often resulting in low self‐esteem and increased risk for depression. This finding illustrates the relationship between self‐esteem and depression.

This result was supported by the study carried out by Hong, Chiu, and Huang (2012), which predicted that smartphones would affect self‐esteem due to the easy access it provides to the users to social networking sites. This finding is congruent with that of the study carried out by Wang et al. (2017), which showed that self‐esteem was negatively associated with adolescent smartphone addiction. Finally, nursing students should be screened for low self‐esteem and depression to guide them for the appropriate treatment selection. Therefore, there is a need to create possible health education programmes and interventions that are appropriate to deal with the addiction to university students and improve their mental well‐being.

### 4.1 Limitation of the study

- This study was cross‐sectional and cannot infer causality.
- Based on the adolescent self‐report measures, future studies should try to collect data from multiple informants (e.g. teacher, peer or parent) to further replicate findings.
- Other interpersonal relationships, such as parent–child relationships or teacher–student relationship, may also influence adolescent smartphone addiction.

## 5 CONCLUSIONS

Based on the results of the study, it is concluded that the prevalence of smartphone addiction is higher among nursing students. The prevalence of depression is at risky levels among nursing students. The statistically significant differences were found between the level of education and smartphone addiction. There were statistically significant differences between gender and smartphone addiction. The statistically non‐significant differences between the number of hours using a smartphone and smartphone addiction were found. There were no statistically significant differences between the purpose of using smartphones and depression, self‐esteem and smartphone addiction. There was a statistically significant correlation between smartphone addiction and depression, and a statistically significant negative correlation was found between depression and self‐esteem. The positive correlation between smartphone addiction and depression is alarming.

## 6 RECOMMENDATIONS

The following is suggested based on the findings of this study:

- The students should reduce the use of smartphones and addiction to it and the priorities in their day‐to‐day tasks.
- Nurses working in addiction should benefit from protective measures for the prevention and effects of addiction not only in the health clinic but also in schools and centres of adolescents wherever they can access.
- Develop prevention nursing programmes for university students and help to raise awareness concerning behavioural addictions and their adverse impacts. Besides, the opportunity of smartphone addiction should be added to the new care plans.
- Develop effective counselling programmes to prevent smartphone addiction of college student by figuring out the level of smartphone addiction and relationship of smartphone addiction with depression and self‐esteem and identify the predictive factors affecting smartphone addiction.
- There is a necessity of raising awareness among nursing students about the biopsychosocial hazards of smartphone addiction.

## CONFLICT OF INTEREST

No conflict of interest has been declared by the authors.

## AUTHOR'S CONTRIBUTIONS

Sayeda M., Mohamed and Mona H., Mostafa: Conception and study design, data collection, analysis, interpretation, manuscript writing, reviewing and revising. All authors read and approved the final manuscript.
